# Study Protocol for the SC-SD4ASA Project: A Self-Care/Self-Development Guidebook for Asylum-Seeking Adolescents

**DOI:** 10.3389/fpubh.2022.736673

**Published:** 2022-07-22

**Authors:** Marjan Mohammadzadeh, Katherina Heinrichs, Laura Pilz González, Christiane Stock

**Affiliations:** ^1^Institute of Health and Nursing Science, Charité-Universitätsmedizin Berlin, Corporate Member of Freie Universität Berlin and Humboldt-Universität zu Berlin, Berlin, Germany; ^2^Unit for Health Promotion Research, University of Southern Denmark, Esbjerg, Denmark

**Keywords:** asylum-seeking adolescents, refugee crisis, intervention development, self-care, self-development, multi-method analysis

## Abstract

From 2015 to 2016, about 1. 3 million refugees arrived in Europe. Half of them were children under the age of 18. The combination of (specially forced) migration and adolescence increase the risk of psychological problems among refugees including asylum-seeking children and adolescents. Therefore, along with the significant increase in the number of refugees, investigating effective ways to improve their health status has grown. The planned project aims to improve self-care and self-development among asylum-seeking adolescents aged 15–18. With the long-term goal of improving wellbeing and quality of life, this multi-method study aims to develop a self-care and self-development intervention guidebook for asylum-seeking adolescents. The SC-SD4ASA project will focus on three main work packages: 1. assessment of needs and knowledge concerning self-care and self-development; 2. development of a guidebook to promote self-care and self-development for asylum-seeking adolescents; and 3. assessment of the guidebook fidelity, reliability, and validity. For the first work package, an intra-triangulation approach including three qualitative methods [in-depth interviews, focus group discussions and photographic means (photovoice)] will be used. The collected data will be analyzed using cross-cultural and multilingual approach to thematic analysis, known as meta-theme analysis. The results of the first stage will be utilized for developing the guidebook in the second (main) work packages. The guidebook fidelity will be assessed based on the National Institutes of Health Behavior Change Consortium fidelity framework in the last workplan. Empowering asylum-seeking adolescents with self-care /self-development skills can help them to sustain their wellbeing and better manage the challenges in their new situation. When successfully implemented, a guidebook will be developed to support all individuals involved in planning, managing, and promoting health among asylum-seeking adolescents that can be used for future self-care/self-development programs in practice.

## Introduction

Today, the refugee challenge is one of the most complicated humanitarian issues the world is facing. Approximately every 2 s, an individual is displaced coercively. Since 2015, nearly 70.8 million individuals have been forced to leave their homes because of war, lack of safety and insecurity. Of these, more than 2 million have chosen Europe as their destination, approximately half of them is under the age of 18. As a consequence, Europe has seen an unprecedented rise in the number of refugees which is known as the “European refugee/migrant crisis” (1).

Keeping these facts in mind, adolescents, in their transition from child- to adulthood, are faced with plenty of challenges to obtain and develop a wide range of skills to establish a foundation for a healthy adulthood (2, 3). When these challenges are combined with (especially forced) migration, two remarkable life milestones collide and increase the risk of psychological and behavioral problems among asylum-seeking children and adolescents (ASCA) (4).

In the past few years, along with the significant increase in the number of refugees, seeking for effective ways to improve their health status and wellbeing has been accelerated. In this respect, many researchers have considered psychological health in ASCA from different aspects, especially its risk factors (5–8).

The outcomes of these studies reveal the excessive rate of mental and psychological issues in this population. For instance, in the European countries, the prevalence of posttraumatic stress disorder (PTSD) among asylum seeking children and adolescents was reported between 20 and 84% (9). Similarly, several studies identified a high prevalence of other mental and psychological problems such as depression and anxiety in asylum seeking children and adolescents (5, 10, 11).

However, despite the broadness of studies reporting the prevalence of psychological problems in asylum-seeking and refugee children and adolescents, there is asubstantial gap regarding the presence of effective, evidence-based educational and therapeutic interventions (12). Generally, the healthcare that ASCA receive is far from satisfactory and there are still many shortcomings (13, 14).

Overall, it has been approximated that more than 90% of asylum seeking children and adolescents that need mental health services, never receive them (15). Thus, the time for decreasing adolescents' psychological problems is limited and any delay in effective actions significantly increases the time span of recovery and severity of the problems (16–18).

On the other hand, the success of broad-based health strategies depends on having a logical and reliable foundation (such as pilot studies) and needs to be integrated with all the available capacities [2019 (17)]. In this regard, the refugees' potential should not be underestimated. According to this view, reframing the nature of the issue in order to enable refugee adolescents through empowering them might be an effective “master plan” in overcoming their challenges (19). The purposeful use of a preventive plan for improving their own lifestyle and wellbeing “by themselves” accounts as one of these approaches (20).

In June 2019, the World Health Organization (WHO) published the “Consolidated Guideline on Self-Care Interventions for Health” (21) as the first of its kind, targeting sexual and reproductive health, and presented it as an important milestone for the WHO. By the same token, self-care/self-development (SC/SD) interventions are considered as the most novel promising and effective strategies aimed to improve different aspects of health and wellbeing. On this note, the concept SC is simply described as a form of care that is provided “for you, by you” to maintain a positive health status and cope with life challenges (22). SD on the other hand, is defined as “having the skills and confidence to take charge of your needs, your everyday roles and responsibilities, and your emotions,” such as enhancing self-awareness (23). Both concepts are self-oriented skills that develop through the communities' capacity such as mutual caring and sharing of health strategies (24).

Empowering ASCA with self-oriented skills (25) such as SC/SD can help them to sustain their wellbeing and better manage life's challenges and adapt to and thrive in the new situation. Although these approaches—with their unique features—seem to have a strong potential to become a part of ASCA lifestyle, there have been no systematic and coherent efforts so far among this population.

The present study investigates potentially protective factors (SC/SD) that have rarely been studied in the ASCA population. The main goal of our Self-Care/Self-Development for Asylum-Seeking Adolescents (SC-SD4ASA) project lies in the development of a new interventional strategy to enhance the psychological and behavioral health among ASCA. This aim goes in line with the current top priory of UNICEF Europe and Central Asia as well as the WHO health strategies such as “triple billion” [one of the WHO comprehensive plan aiming to improve the health of billions of people by 2023 (26)]. Developing an intervention program for ASCA to improve their lifestyle and consequently, their wellbeing and quality of life, based on their needs and situations, is a novel approach paving the pathway for years ahead in the bridge among primary health care, host societies and health delivery.

### Study Objectives

With the mid- and long-term perspective of improving asylum-seeking adolescents' psychological and behavioral health using their potential abilities, the overall objective of the study is to develop a SC/SD guidebook which will be effective in promoting psychological health (specifically anxiety and stress) and quality of life among asylum-seeking adolescents in Germany. This guidebook, in form of an activity book/personal journal, will be based on their specific needs, cultural background, the culture of the host country and resource accessibility. Based on this ground notions and aims, our study will focus on the following three specific objectives:

1. The FIRST objective is to study self-care and self-development needs assessment among asylum-seeking adolescents in Germany through three different perspectives: phenomenology, cultural and gender perspectives. The increasing of knowledge and information about the current SC/SD practices and experiences among asylum-seeking adolescents is included in this objective.
2. The SECOND objective is to develop an interventional guidebook aiming at improving the psychological health, coping mechanisms as well as social and emotional adjustment of asylum-seeking adolescents through a multi-stage study. This guidebook will be designed based on an in-depth and people-centered health and wellbeing approach that is supported by the key principles of ethics, culture and gender dimension.
3. The THIRD objective is to finalize the guidebook development by assessing its fidelity and inter-rater reliability with the collaboration of a related interdisciplinary panel. Fidelity accounts as a vital element for any behavioral intervention to ensure that the outcome is in accordance with the set objectives and addresses both internal and external validity (27).

## Methodology

**Figure 1:**
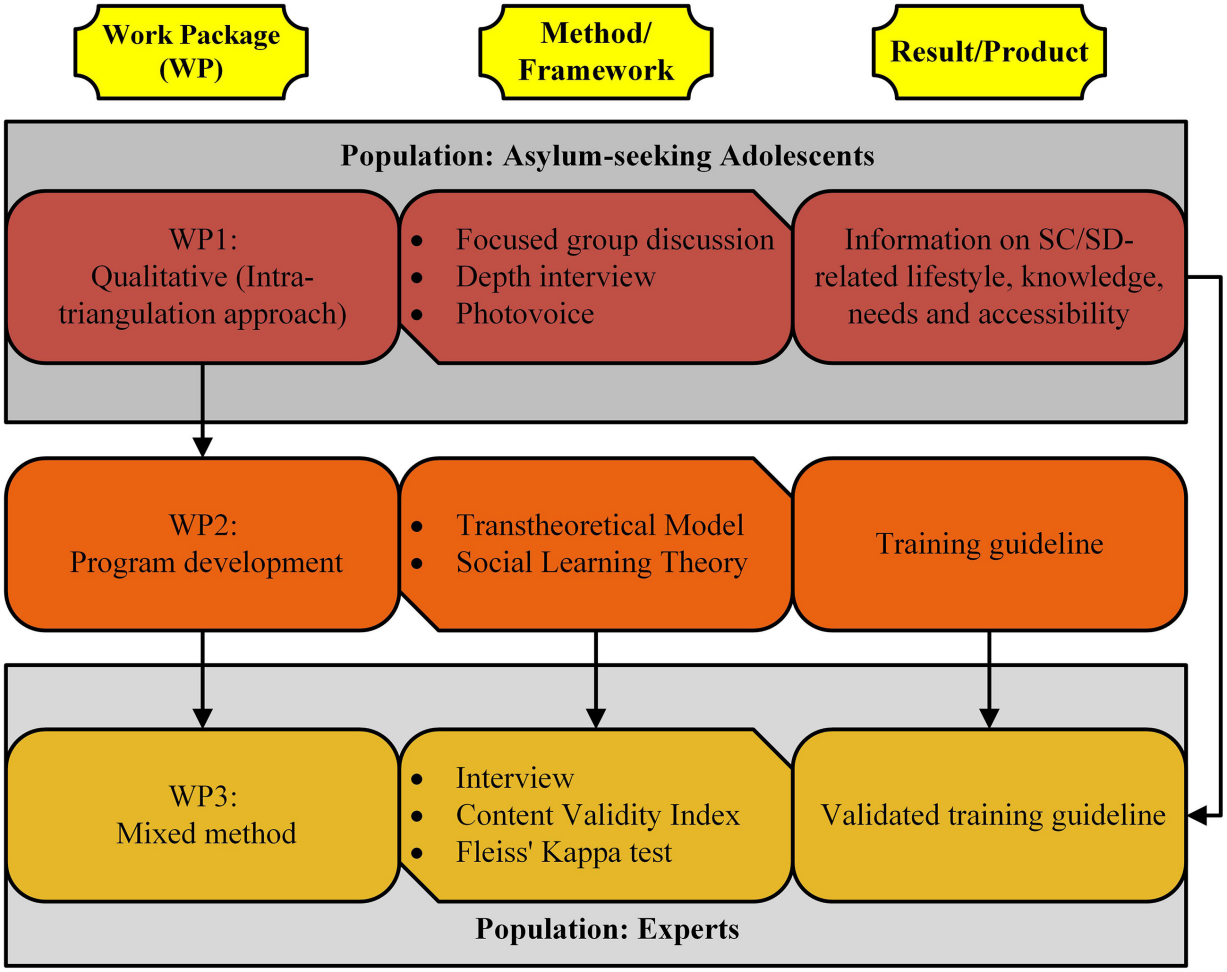
Overview of the SC-SD4ASA study work packages.

Overall, the SC-SD4ASA study will follow a multi-method approach, including intra-methods of qualitative and quantitative studies (Figure 1), directed to address the specific research objectives outlined above. Successful implementation of this project depends on the accurate implementation of these approaches; all planned approaches and methods need to be carefully established and thoroughly used.

The multi-method approach generates synergetic effects to improve the quality of the results and guideline development. Through the implementation, process it is possible to facilitate the practical integration of the final results. The SC-SD4ASA methodology is briefly divided in the three work packages (WP) as follows:

### WP1: Assessing the SC/SD Knowledge and Needs Among ASCA in Germany

To achieve this goal, an intra-triangulation approach (28) including three qualitative datasets from in-depth interviews, focus group discussions and photographic (photovoice) data will be combined. This will allow for the exploration of knowledge, needs and resources concerning SC/SD in general quality of life as well as emotional health. Further, SC/SD learning and practicing opportunities (formal and informal), already offered to the participants, will be identified. The data collection protocols can be found as the Supplemental Materials.

The strength of combining the text and imagery datasets offers an enriched dataset based on a broad range of perspectives to assess information about “what, why and how” participants feel, think and act regarding SC/SD. As well, the triangulation data collection enhances the comprehensiveness, validity and reliability of the study (29). On the other hand, this method gives rise to a large amount of data and takes a lot of time and resources and hence, it may not be cost-effective. Therefore, it is crucial to determine when to stop which will occur at a point where data collection reaches a saturation point in which no new insights are collected (30).

#### Participants and Sampling

The participants will be selected from eligible asylum-seekers aged 15–19 years, in German public schools, that are willing to participate in the project (purposive homogeneous sampling). Demographic and socio-demographic sensitivity such as gender and ethnicity/culture will be carefully considered in the sampling process. Adolescents in the early stage (aged 12–14 years) will not be included in the data collection as their daily plans are mostly influenced by their families, especially in the Middle East culture (18, 31).

#### Data Collection and Data Analysis

Data will be collected and analyzed using the following approaches and methods:

1. The metatheme analysis (32, 33) method will be used for collecting and analyzing the data from interviews (estimated sample size 30) and focus group discussions (4–5 groups with ~20–25 participants in total). Metatheme analysis is a new qualitative approach which fundamentally focuses on themes that occur across different datasets (33). In this study, this accounts for different languages and/or cultures. With the participation of refugee adolescents from various countries, this method will be applied to interviews or group discussions that explore knowledge, experience, and educational needs related to self-care and self-development. The data collection protocols, including face to face in-depth interviews, focus group discussions, and photovoice [see Additional File 1], will be developed carefully to prioritize the systematic aspects of the study, expand ideal sampling strategies, and establish appropriate protocols to address the linguistic and ethnographic knowledge of each dataset (34, 35). To do so, we will also invite linguistics experts with long-term ethnographic experience to our data analysis team to ensure adequate capacity of the identified themes in each dataset.

**Figure 2:**
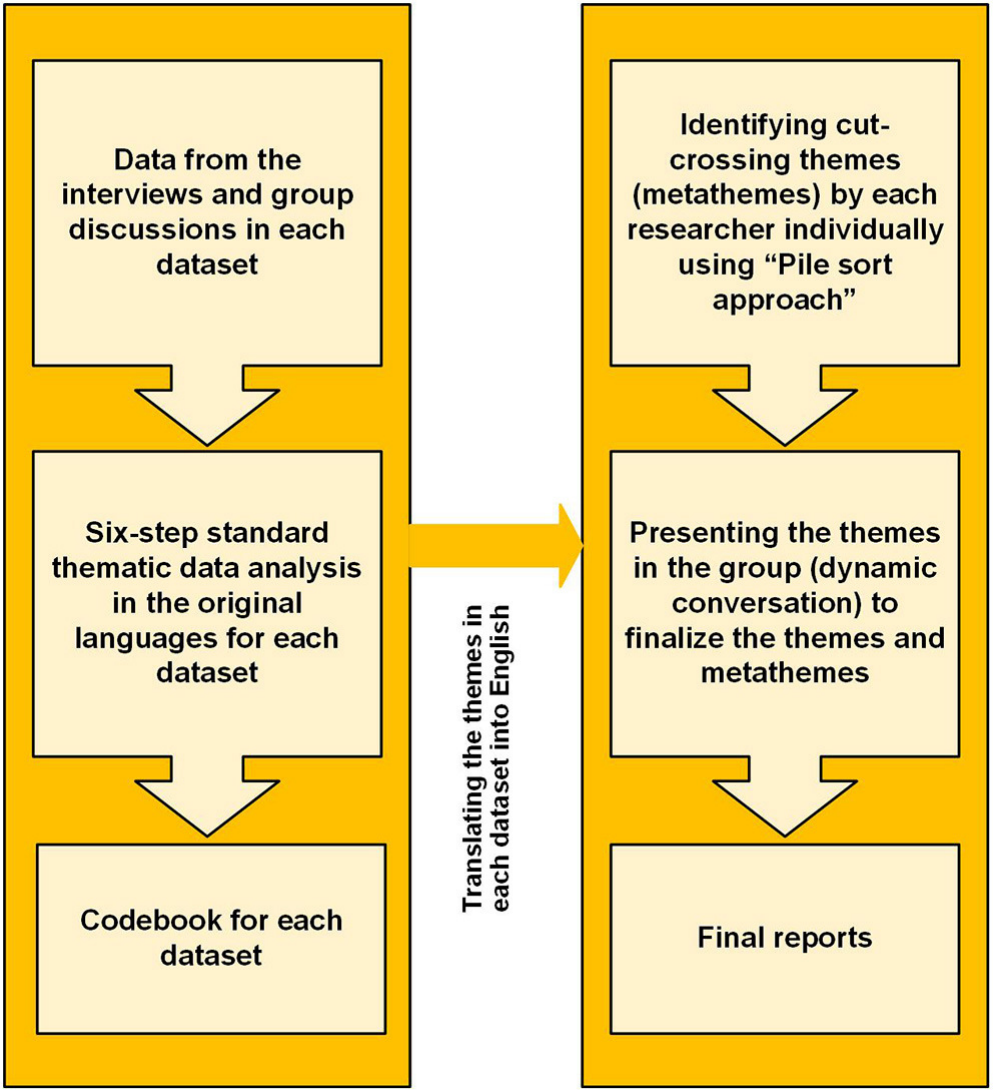
Metatheme analysis process.

For the data analysis (Figure 2), the interview and group discussion sessions will be audio-recorded and transcribed in their original language. All transcribed records from the interviews and group discussions will be analyzed by following the six-step standard thematic analysis (36) by two independent researchers. The thematic analysis will be done in the original language, as translating the data in the initial stages may increase the risk of losing the depth of concept and meanings of words and statements. The process of coding will be documented in the form of a codebook. Finally, the original transcripts will be double-checked to ensure transparency and accuracy in the analysis. Moreover, trustworthiness will be achieved by requesting a rich description of concepts in the interviews and group discussions and facilitating member checking. Repeated meetings with the research team will also contribute to the accuracy of the data analysis.

In the second step of the data analysis, all non-English codes and themes will be translated to English and back to the original language by another translator to ensure their accuracy. The translated themes from both datasets will be sorted into piles by at least two independent researchers separately and suggested cross-cutting metathemes will be identified. Then, each researcher will present and explain their sorting process and list of themes to the research team. The presented results will then be discussed in a dynamic way in the research team and thus, the final overarching metathemes will be defined. This systematic and dynamic sorting process of themes and overarching metathemes enables the collected data to be contextualized and its depth not to be lost in translation, but instead, be consolidated into crosscutting metathemes (34).

Data reliability will be achieved through utilizing efficient interviewing and mediating methods and accurate transcription as well as translation (37). All researchers conducting interviews and focus group discussions will follow an interview guideline and will attend a training prior to the data collection and assessment. In addition, consistent transcription rules will be applied to improve comparability of the records.

1. Visual data (photovoice approach, ~15–20 photographs) will be collected according to the SHOWeD method (What do you see here? What is really happening here? How does this relate to our lives? Why does this problem or situation exist? How could this image Educate others? What can we do about the problem or situation?) (38) [see Additional file 1]. Additionally, the photovoice approach will allow us to gain a deeper understanding of the participant's daily life. This qualitative method was firstly introduced by Wang and Burris in 1994 (39, 40) and centers around the notion of sharing ideas and stories through photographs. In this way, participants can identify and highlight issues of importance to them and capture them in a “snap-shot” which then, helps to get a deeper view regarding the issue under study (38). Using images in conjunction with other kinds of data, such as dialogue and voice (as we are doing with focus group discussion and in-depth interviews), can lead to a comprehensive and rich range of data and information, addressing complex health and social issues (41).

We will collect some background information like age, country of origin, school level, and the number of years they have been out of the home country. Moreover, the outcomes of our qualitative data related to the diversity and cultural/historical background factors will be the important guide in designing the activities or even pictures of this guidebook. This data will be used only for scientific purposes by the research team, and no one out of the team can access the participants personal information or identify.

To obtain more comprehensive data for this study, each participant will be placed in only one of the interview or discussion groups. However, due to the different nature of photography data, the participants in this group can also be involved in interviews or group discussions. It is noteworthy to highlight that in qualitative research, the approximation of sample size is contextual and dependent on the scientific model under which the study is taking place and is therefore somewhat flexible (42). However, the common idea of a sample size in qualitative researches is to reach “data saturation” point (30).

### WP2: Developing the SC-SD4ASA Intervention Guidebook in Form of a “Self-Care and Self-Development Personal Booklet/Journal”

Developing a new evidence-based educational and interventional program in community health needs a systematic approach starting with determining the scope and goals of the addressed issue, followed by devising its characteristic factors including population, design and outcomes. These stages need reliable evidence, comprehensive systematic review and the outcomes of previous studies (e.g., pilot studies). Each stage also needs to be divided into logical milestones and each milestone should be scientifically evaluated before going to the next one. For SC-SD4ASA, the Social Learning Theory (LST) (43) and the Transtheoretical Model (44) will be followed as theoretical framework.

Overall, using data and information obtained during the WP 1 assessing knowledge, needs, existing resources, and ethical considerations related to different groups of participants, an evidence-based SC-SD4ASA guidebook will be developed including logical and practical self-oriented activities to address the main objective. A detailed description of the SC-SD activities in the guidebook is not possible at this stage, since it will rely on evidence and information collected in WP 1.

However, relaxation techniques such as deep breathing to reduce anxiety and stress, daily High-Intensity Interval Training (HIIT), girls/boys' night in/out (considering their age and culture, with parental supervision), forming sports teams as well as sharing skills e.g., arts and crafts can be introduced. Furthermore, the establishment of a local library to share books and practices of self-love/self-worth are some ideas which can potentially be considered as SC/SD activities for the guidebook.

Along with the main sections of the detailed activities, the SC-SD4ASA will contain an introduction on SC/SD history and activities, the importance of SC/SD-based activities for youth, especially for ASCA as well as the objectives of the guidebook. The details of implementation of the activities, suggested evaluation methods for training sessions and answering options to common questions are also planned to be included. Each session will include presenting SC/SD goals, practicing and discussing possible activities while also leaving room for questions and answers.

**Figure 3:**
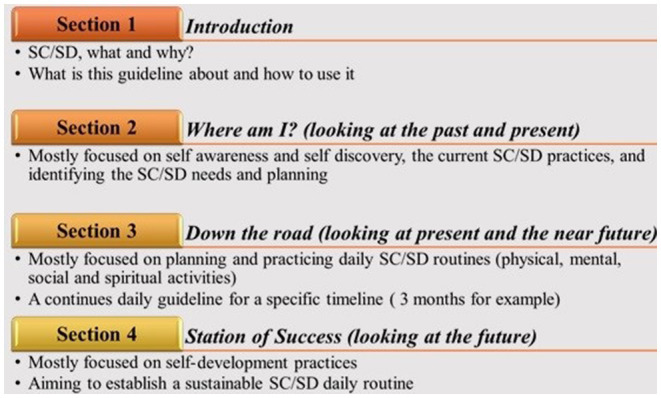
Overview of the guidebook sections.

Due to the interdisciplinary nature of this intervention approach, the evolutionary process will involve a panel of related experts such as psychologists, behavioral consultants, teachers, social workers, etc. The guidebook will consist of 12 distinct sections (each representing a month of year) including self-care and self-development related activities, personal planers, experts' notes, games, etc. In each section, the topics will start with “current” issues, situations, abilities, and facilities around the self (last month self-evaluation), followed by “moving ahead” to new ideas, practices, and achievements (plans for the current month). Finally, the guidebook will focus on the activities which are more related to the “adolescent's future,” such as starting to learn a new skill [next month(s) plans]. So, the SC-SD4ASA guidebook will contain 4 main concepts, including: 1. Self-care and self-development definitions, importance, and examples 2. Where am I? (Looking at the past and present) 3. Down the road (looking at present and the near future) 4. Station of Success (looking at the future). It also comes with the suggestions of a “how-to-use” prerecorded workshops and multi-language YouTube/TiktoK videos which would be supported by social media applications and platform pages and groups (Figure 3).

The final product will be in the form of an informative guidebook, including a personal planner and a self-assessment activity book. In addition, multilingual tutorials and workshops will be created and shared on popular social media and platforms among this specific target group.

### WP3: Assessing the Fidelity, Validity, and Reliability of the SC-SD4ASA

It is vital for any behavioral intervention to ensure that the outcome is in accordance with its specific objectives and addresses both internal and external validity (27). As such, the SC-SD4ASA fidelity protocol will be developed based on the National Institutes of Health Behavior Change Consortium (NIH BCC) fidelity framework which includes five domains: study design, provider training, treatment delivery, treatment receipt, and treatment enactment (45). In this study, the fidelity protocol in each domain will be designed and applied after completing the final version of the guidebook.

In addition, to assess the guidebook's content validity, the Content Validity Index (CVI) approach will be used (46). In this regard, the guidebook will be reviewed by a panel of experts in children and adolescent psychiatry and psychology, children and adolescent education, and community health. Using a questionnaire with responses ranging from 1 (not relevant) to 4 (highly relevant), the Item-level Content Validity Index (I-CVI) and the Content Validity Index for Scales (S-CVI) will be calculated using the following formulas (46):


$$\begin{array}{l} I-CVI=the\;number\;of\;experts\;giving\;a\;rating\;of\;either\;3\;or\;4\\ \;/the\;total\;number\;of\;experts\\ S-CVI=the\;sum\;of\;I-CVIs/\;the\;number\;of\;items \end{array}$$

According to the literatures, the minimum I-CVI of 0.77 (for 6 to 10 experts) and the minimum S-CVI of 0.90 are requested for an excellent content validity (values range is from 0 to 1) (47). As well, inter-rater reliability of SC-SD4ASA will be evaluated by two independent experts using the Fleiss' Kappa reliability test (48). The nature and variety of activities associated with SC/SD, including the simplicity, not requiring a specific environment for the implementation, and self-centered implementation, potentially increase the chance of the project's successful implementation, even among difficult population groups.

## Expected Results and Future Plans

At the end of WP1 of the study, it is expected to have acquired information on SC/SD-related lifestyle as well as knowledge, needs and accessibility concerning SC/SD among asylum-seeking adolescents in Germany. Finishing WP2, it is expected to reach novel sets of evidence-based and reasonable SC/SD activities designed specifically based on the data declared by asylum-seeking adolescents themselves. The guidebook can be made available to the adolescents (online and in hard copy), through the project website, institutions related to refugee families and adolescents, such as schools, after obtaining the requested permissions.

**Figure 4:**
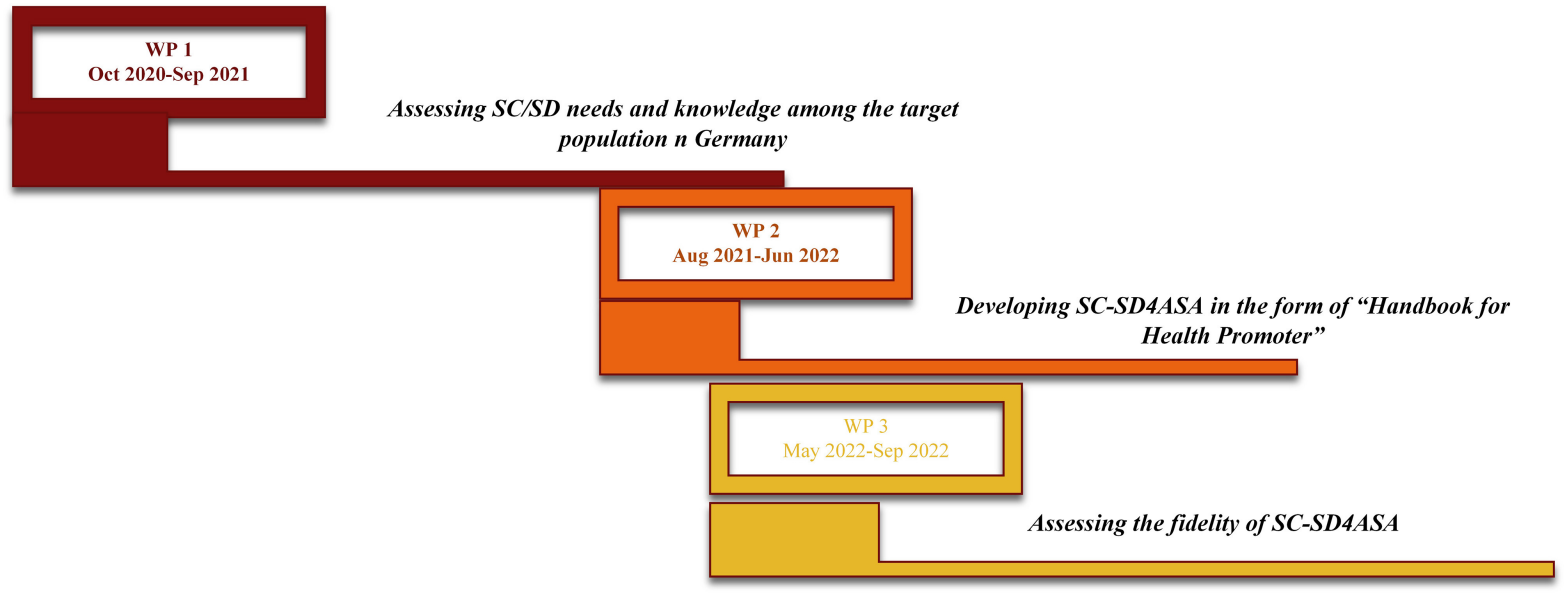
The SC-SD4ASA study timeline.

Finally, utilizing the results obtained beforehand is expected to lead us to the study's ultimate objective as developing the first ready-to-run guidebook designed to use ASCA's own potentials to improve their mental and behavioral health (Figure 4). Finalizing this guidebook can be a significant step forward to allow ASCA to take control over their lives and gain access to broadened abilities needed in the pathway to successful adulthood.

The SC-SD4ASA guidebook can provide an opportunity to shift from ambition to accessible health targets for all, and most valuable of all, it can have an affirmative effect on the lives of this vulnerable population i.e., ASCA. Although the study will be conducted in Germany, it will be designed to be adjustable and have the potential to be used in other host countries.

Implementing and evaluating the guidebook among refugee adolescents is not included in the current project study. However, translation of the guidebook into the common languages of the refugees in Germany (such as Persian, Arabic, German, and due to the recent situation Ukrainian) as well as assessing the effectiveness of the guidebook on refugee adolescents' quality of life, emotional health, and coping skills through a community-based pragmatic controlled trial are defined as objectives for future projects.

## Discussion

The SC-SD4ASA study relates with a novel approach regarding asylum-seeking adolescents' wellbeing by addressing one of the most important concerns of organizations and host countries involved in refugee affairs: improving mental and behavioral health among ASCA. Here, the issue is more specific: developing a self-care (SC) and self-development (SD) intervention that improves the emotional health and quality of life among asylum-seeking adolescents in Germany. Although other approach to improve the health and wellbeing of asylum-seeking adolescents exist (49–52), to the best of our knowledge this is the first intervention program specifically designed to harness these youths' own potential to enhance their health status.

There is a substantial interdisciplinary aspect and gender dimension in the research content of the study. SC-SD4ASA is a purposeful attempt to organize representatives from several scientific disciplines including psychology, social and behavioral sciences, health education, health policy, and diversity/culture. It aims to design and shape the research process, expand the understanding of the study factors and objectives, manifest different perspectives of the study's nature and obtain its maximum short- and long-term goals. Due to the interdisciplinary and multi-activity nature of SC/SD approaches, it is important for health systems to recognize their significance and effectiveness. Additionally, SC-SD4ASA study is strongly influenced by gender dimensions as there are significant differences in needs, attitudes and accessibility toward SC/SD activities between male and female adolescents. Failure to consider these differences can completely divert the project from its goals or at least attenuate its effectiveness considerably. Therefore, the activities will be designed for each gender separately to achieve gender equality, although in some cases there may be overlaps.

This research will use state-of-the-art methods e.g., three-source inter-method data collection for obtaining unique and rich data from the study's population point of view. Therefore, allowing us to develop an innovative plan in the search of strategies for enhancing psychological and behavioral health among ASCA through their own experiences and perceptions.

Nevertheless, there are some potential limitations in that asylum seekers are a vulnerable group owing to their traumatic background and current situation which needs particular attention. Although many SC/SD activities are expected to cost nothing/little money, it cannot be disputed that they have their own costs, some of which are traditionally known as government responsibilities. Therefore, the hidden costs of shifting responsibility from governments to individuals should be considered carefully. The sensitivity of this issue becomes clearer when it comes to knowing that ASCA often do not have a financial income. Further, because of the specific circumstances of families, getting help from parents is potentially limited. Therefore, it is not likely that the youngsters get any help by their families in this regard.

Besides, some of these activities have non-financial costs e.g., time. It is also notable that following these approaches does not mean to disclaim responsibility from authorities or governments for quality health services, which could be considered as a risk factor of causing more problems or worsening them. However, the cost of NOT taking care of one's self in the long-term far outweighs the short-term costs. With precise screening of capabilities on the one hand, and the barriers and limitations on the other, the above constraints can be overcome. The final outcomes of the SC-SD4ASA study can be a starting point for further research, especially concerning the impact of the designed intervention on adolescents.

## Ethics Statement

The project's ethics approval is obtained from the Research Ethical Committee of Charité Universitätsmedizin Berlin (number EA2/087/21) on 08.06.2021. Written informed consent to participate in this study was provided by the participants' legal guardian/next of kin.

## Author Contributions

MM: conceptualization, investigation, methodology, project administration, and writing—original draft preparation. LP and KH: investigation, methodology, and writing—review and editing. CS: conceptualization, methodology, supervision, and writing—review and editing. All authors substantively revised the manuscript and have approved the final submitted version.

## Funding

This project has received funding from the European Union's Horizon 2020 research and innovation program under the Marie Skłodowska-Curie grant agreement No 888607. The funding body played no role in the development of this article. The study protocol has undergone peer-review by the funding body.

## Conflict of Interest

The authors declare that the research was conducted in the absence of any commercial or financial relationships that could be construed as a potential conflict of interest.

## Publisher's Note

All claims expressed in this article are solely those of the authors and do not necessarily represent those of their affiliated organizations, or those of the publisher, the editors and the reviewers. Any product that may be evaluated in this article, or claim that may be made by its manufacturer, is not guaranteed or endorsed by the publisher.

## Supplementary Material

Additional File 1 Data collection guideline for WP1 (interview, group discussion, and photovoice), pdf file.
