# Supplementary material for: Study Protocol for the SC-SD4ASA Project: A Self-Care/Self-Development Guidebook for Asylum-Seeking Adolescents
Source: Front Public Health. 2022 Jul 22;10:736673. doi: 10.3389/fpubh.2022.736673 (PMC9353327; doi:10.3389/fpubh.2022.736673)
Supplement: Additional File 1 — Data collection guideline for WP1 (interview, group discussion, and photovoice), pdf file. [file Data_Sheet_1.DOCX]

**Data Collection Guidelines**

***Notes for the readers:***

*Interview/discussion guide and questions can change during the data assessment phase which is quite common in qualitative research. Therefore, this is only one possible version of the data collection guidelines.*

*Before starting the interview/discussion, we will explain the participants the general concept of Self-care (SC) and Self-development (SD), briefly.*

**Interview Questions**

**Introduction**

My name is (…) and I work as a researcher at the Institute of Health and Nursing Science, Charité - Universitätsmedizin Berlin. As part of our project on Self-care (SC) and Self-development (SD) among asylum-seeking adolescents in Berlin, we would like to ask you about your ideas, experiences and activities regarding these two concepts. The aim of our study is to create a SC/SD training with specific activities to help people of your age and in a similar situation to stay healthy, both physically and emotionally. I would like to welcome you to the study and thank you for your time, we really appreciate it.

This interview will take about 30-45 minutes and I will be asking you a few personal questions to get to know you a little and then questions regarding your familiarity with SC and SD as well as any possible experiences with activities in this regard. Here you can also share recommendations and ideas about creating such a workshop. Remember, this is a safe space and that there are no “wrong” or “right” answers and everything will remain anonymous and confidential. Also, I would like to remind you that your participation is voluntary, which means that you can drop out at any time. If there are any questions you would rather not answer just let me know, you are not obliged to answer. The interview will be transcribed, and you can ask for a review of the transcript or that we delete your data.

Do you have any questions before we start?

As such, do you agree that our conversation will be recorded?

First, I would ask you to repeat your name and date of birth. How old were you when you left your country (name of the country)? (not recorded)

**Part 1: Knowledge**

1. Thank you for participating in this interview. Let us start with a general question. What do you do to stay healthy … in your body … in your mind?
2. What ideas do you have, what could SC-SD be?
   1. Have you ever heard of self-care (SC) and self-development (SD) concepts? Where? When?
3. Regardless of what you have or have not heard about SC and SD before, what is your own understanding of them? Can you describe them in a few sentences?
4. Can you think of some examples of SC/SD activities? Would you mind sharing them with me?
5. What do you think are the differences and similarities between SC/SD activities? Or do you think they are basically the same?

***Examples of Probing Questions:***

- Can you be more specific?
- What do you mean when you say […]?

**Part 2: Experience**

Let us focus on you as a migrant adolescent.

1. What do you do for your own SC/SD? What is the priority and importance of these activities in your daily routine?
2. How do you feel when doing these activities?
3. If you do not have any SC/SD activities, could you imagine setting up SC/SD goals? Which ones?
4. Do you have any SC/SD long-term goals, for example for this year?
5. You have immigrated to Germany from your country of origin with a specific history, culture, and values. Do you think having this background and experience can affect your SC/SD goals, plans and activities? How?
6. Do you think gender affects doing SC/SD activities? How come?
7. Have you experienced something/met someone that motivated you into doing these activities? Can you tell me more about that experience?
8. In your opinion, what are the most important barriers for doing SC/SD activities for you and your peers as a migrant adolescent?
9. Have you experienced any of these barriers?

9.1. -If yes, would you mind sharing which ones and how you have experienced it?

***Example of Probing Questions:***

- Why do you name this activity as SC/SD?
- Can you be more specific? Any example?
- Tell me more about that.
- How did you feel about that?
- What do you mean when you say […]?

**Part 3: Need Assessment**

1. What skills/knowledge would you say you need as a teenager with a migration background regarding SC/SD activities?
2. In what way do you think gender has an influence on these needs? Would you say you as a teenage female/male have different SC/SD needs; how come?
3. Imagine that someone tells you that you can attend a training course related to SC/SD. What do you think this course should include? What training will help you to do SC/SD daily activities
4. How do you think this program could be run so that it is beneficial for you? Probing: Individually or in groups, with/without practical exercises? Depending on answer add probing here: how should these groups/ exercises look like?
5. As a final question, if it is possible for you to participate in such a training course, do you think you would like to spend part of your daily time participating in it? Why?

***Example of Probing Questions:***

- Can you be more specific?
- Tell me more about that.
- What do you mean when you say […]?

**We are almost done with the interview. But we would like to ask you to add a question or explain an experience related to SC/SD which we may not have mentioned during the interview.**

Is there anything else you would like to add?

To conclude this interview, I will tell you 4 sentences. Please complete them with a shortlist of about 5 words. Of course, you may have already mentioned them in your answers.

1. The activities that I would like to do, to have a better mental health ……
2. The activities that I would like to do, to have a healthier body …………….
3. The activities that I would like to improve my abilities/skills to have a better life or future …….
4. To perform these activities better and more effectively, I need ……

Thank you so much for your time.

**Group Discussion Questions**

***Notes for the readers:*** *before starting the discussions, we will have an introduction session, same as the interviews.*

- What is your definition of self-care /self-development (SC/SD) activities? Have you ever heard of self-care? If yes, can you describe in your own words what you think it is?
- What is the role of SC/SD activities in migrant adolescents’ life?

If you think about your peers without a migration background, do you think there are any SC/SD activities specific to you with a migration background? What would these be?

- What can hinder you from doing these kinds of activities as a male/female migrant adolescent? Can you give an example, preferably from your own or your friends/family experiences?
- In your opinion, what support, facilities or training would you need to get the best skills regarding SC/SD activities?
- Imagine that someone tells you that you can attend a program related to SC/SD. What do you think this course must include? What training will help you do your SC/SD daily activities?
- Is there anything else you would like to add before we end?

**Photovoice Protocol**

***Notes for the readers:*** *Participants will get a detailed face to face (online) information about the photovoice. This is only a summary of the details.*

**Main Objective:**

Self-care (SC) and self-development (SD) activities among immigrant adolescents in Germany

**Topics:**

- SC/SD among immigrant adolescents: Definition: What is SC it for me? What is SD for me?
- SC/SD among immigrant adolescents: Benefits: What is good about SD for me? What is good about SC for me?)
- SC/SD among immigrant adolescents: Activities: What are SC activities? What are SD activities?
- What SC activities do I like? What SD activities do I like?
- SC/SD among immigrant adolescents: Resources: What do I need to be able to do the SC/SD activities?
- Self-care/self-development among immigrant adolescents: Barriers/limitations: What can hinder me from doing SC activities?
- SC/SD among immigrant adolescents: Training: What/who can support/teach/train me to get the best skills for the activities?

**Time and Location:** Anywhere, anytime, no limitation (please get the required permission(s)in advance, if applicable)

**Numbers of the photos:** At least 1, up to 10.

**Tool:** Camera, Mobile phone, webcam etc.

**Photos Characteristics:** Related to at least one of the study topics; clear.

**Important note:** We will make sure that all personal identification will be removed from the photos by the main researcher. However, if it is possible for you, remove/hide any personal identification details before sending the pictures. This way we can work together to maintain your anonymity.

**Please attach the following explanation regarding each of your photos:**

- A short explanation about the photo. How would you describe it if you had to explain the photo to someone that could not see it? You can select a name for each of your photos.
- What is the most important characteristic of this photo?
- What do you try to express/convey using this photo?

^*^Please send us this information though text, voice or video records.
